# The effect of classical foot massage on insomnia and anxiety in preeclamptic pregnant women: a randomized controlled study

**DOI:** 10.1590/1806-9282.20230744

**Published:** 2024-02-26

**Authors:** Ayça Şolt Kırca, Nurdilan Şener Çetin

**Affiliations:** 1 Kırklareli University, School of Health Science, Department of Midwifery - Kırklareli, Turkey.; 2 Firat University, School of Health Science, Department of Nursing - Elâzığ, Turkey.

**Keywords:** Massage, Insomnia, Anxiety, Pregnant women

## Abstract

**OBJECTIVE:**

Preeclampsia is one of the most common complaints during pregnancy. Preeclamptic pregnant women may experience insomnia and anxiety.

**METHODS:**

This study was a randomized controlled trial with 71 preeclamptic women. In the experimental group, a foot massage was done for 3 days in a week. In the control group, any applications were not done. These groups were assessed for insomnia and anxiety levels.

**RESULTS:**

In this study, it was found that classical foot massage significantly reduced (12.45±5.74 vs. 33.4±6.41) insomnia and anxiety compared with the control group (18.8±6.44 vs. 39.19±8.31, respectively, p<0.05).

**CONCLUSION:**

The classical foot massage can effectively decrease insomnia and anxiety symptoms.

## INTRODUCTION

Preeclampsia (PE) is among the critical reasons for maternal morbidity and mortality all over the world^1^ ^,^ ^2^. In a systematic review of epidemiological studies, the incidence of PE was seen in 0.2-9.2% of pregnancies^3^. While PE makes a healthy pregnancy risky, the expectant mother has to think about a serious disease that threatens both her and the fetus. Accordingly, feelings, such as fear, helplessness, and uncertainty about the progress of pregnancy, may have negative effects on the emotional state of the woman.

Women’s sleep is adversely affected by factors such as increased fetal movement with changes in hormone levels and respiratory and cardiovascular system functions during pregnancy, pressure on the bladder from the growing uterus, and nocturia^4^. Multiple sleep disorders in pregnancy manifest themselves as poor sleep quality, insomnia, and sleep-disordered breathing^5^ ^,^ ^6^ ^,^ ^7^. In their meta-analysis, Lu et al., found a correlation between general sleep disorders and PE^4^, and that maternal psychosocial deterioration (depression, anxiety, and post-traumatic stress disorder) increased in women with PE^8^.

Pharmacological methods, which are generally used to reduce anxiety and insomnia symptoms, may adversely affect the pregnant woman and the fetus^9^. For this reason, non-invasive, low-cost, and uncomplicated methods should be preferred to reduce anxiety and insomnia that can develop due to PE^10^. Massage improves the sleep quality of the individual by relaxing the muscles and reducing stress and anxiety^11^ ^,^ ^12^ ^,^ ^13^. It stimulates cutaneous and subcutaneous sensory receptors, thereby helping venous return and lymphatic flow. It also helps reduce the feeling of pain and remove lactic acid from the muscle fibers, thereby reducing the individual’s fatigue and anxiety^14^. Many studies proved that classical foot massage reduces anxiety and insomnia in individuals^13^ ^,^ ^14^ ^,^ ^15^ ^,^ ^16^ ^,^ ^17^ ^,^ ^18^. However, there is no study on the application of classical foot massage to reduce anxiety and insomnia symptoms in preeclamptic pregnant women.

This study was planned as a randomized controlled trial to reduce insomnia and anxiety symptoms by applying classical foot massage to pregnant women diagnosed with PE and hospitalized.

## METHODS

A prospective, randomized controlled trial design was used. Pregnant women hospitalized in the obstetrics ward of Elazıg Fırat University Hospital between June and December 2022 with a diagnosis of PE and who wanted to participate were included in the study.

Inclusion criteria were as follows: diagnosis of PE, age range of 20-40 years, no diagnosis of a pre-pregnancy sleep disorder or anxiety, having a score of 37 or higher on the state anxiety (STAI-2) scale, and insomnia severity index (ISI) scores of ≥8.

Exclusion criteria were as follows: pregnant women who stayed in the hospital for <3 days, had received treatment for sleep disorders or anxiety, had a chronic disease except for PE, lost their baby during the study, had a bone deformity on foot, had any obstacles to the massage application as decided by an expert, and were allergic to baby oil, vaseline, or any product.

### Randomization

**Figure 1.**
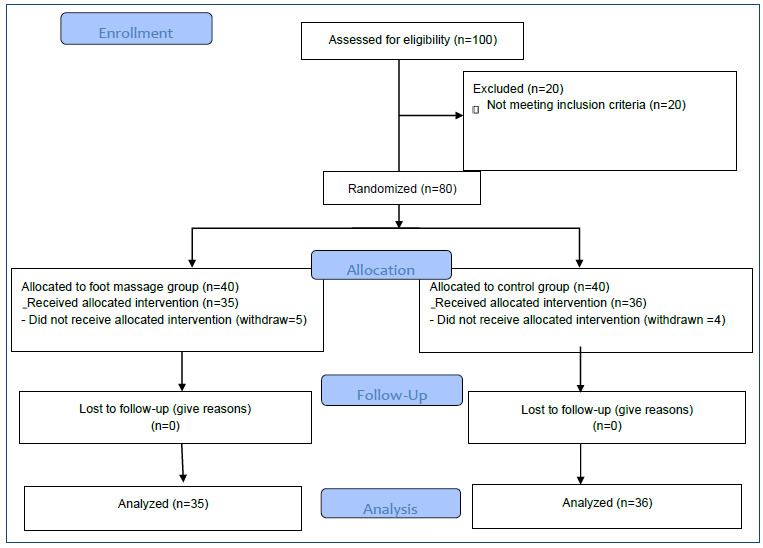
Consort flow chart

A review of the literature indicated that there was no study on the application of classical foot massage to reduce anxiety and insomnia in preeclamptic pregnant women. However, it was reported by some studies that classical foot massage reduced anxiety and insomnia symptoms^13^ ^,^ ^17^ ^,^ ^18^ ^,^ ^19^ ^,^ ^20^ ^,^ ^21^. The number of subjects to be included in the research was calculated using the G*Power software, and the COHEN standard effect size was assumed as 0.70^22^. Accordingly, the sample size was calculated as at least 68 individuals (α=0.05, 1-β=0.80, effect size=0.70, Figure 1)^23^.

### Ethics approval

Approval for this study was obtained from the Fırat University Non-Invasive Research Publication and Ethics Board, Fırat University Hospital (Reference number: E-31970446-050.05-187647, Date:03/06/2022) before the data were collected. The study was registered at www.clinicaltrials.gov before the data collection process was initiated. All procedures were in accordance with the 1964 Helsinki Declaration of Human Rights and its subsequent amendments or comparable ethical standards. The subjects were free to discontinue their participation at any stage.

### Measures and instruments

#### Participant information form

This questionnaire includes 21 questions about sociodemographic and sleep characteristics during pregnancy.

#### The insomnia severity index

This seven-item scale was designed to identify the level of insomnia symptoms^24^ ^,^ ^25^. Boysan et al., conducted the Turkish reliability and validity study of the scale in 2010^26^. Boysan et al., found Cronbach’s α as 0.79. The alpha value in this study was calculated as 0.79.

#### The State Anxiety Inventory

This inventory was developed by Spielberger et al^27^. Öner and Compte adapted the scale to Turkish culture and conducted its reliability and validity study^28^. In the Turkish adaptation of the scale, Cronbach’s alpha was found to be between 0.83 and 0.92^28^. The alpha value was found as 0.85 in this study.

### Data collection

We recruited pregnant women who fulfilled the research criteria and informed them about the research procedures before the application. Accordingly, they knew that they could quit the research when they wanted, and they delivered a consent form. To prevent bias, a nurse/midwife who worked in the department on the day of data collection and was not included in the research helped participants fill out the participant information form and the ISI and STAI scales. Both the midwife or nurse who helped participants complete the scales and the participants were given information about the study steps. However, they were not informed about the purpose of the massage application, who it would be applied to, and what it would do. Thus, as midwives/nurses other than the researcher who performed the application were blinded, the placebo effect was tried to be minimized by preventing the psychological relief of the women in the study. Participants were randomized to experimental and control groups on a website. These groups were recorded by the researcher in a list.

### State Anxiety Inventory, the insomnia severity index, and the participant information form measurement

Participants filled out the questionnaires in 20-25 min during face-to-face interviews. After the 3-day intervention phase, all participants completed a post-intervention ISI and STAI scale by the nurse/midwife who was not included in the research.

### Experimental group

Before they went to bed, pregnant women were given a massage for 20 min, including 10 min for each foot, in each session for 3 days in a week (60 min in a week) by using a chronometer^29^.

During the data collection process, all participants were given a massage by the same researcher who had received basic massage education. Participants took a comfortable position and were advised not to speak unless necessary during the massage session. Afterward, they were given a massage with baby oil or vaseline cream. The massage was applied to five parts of the foot, namely, the back of the foot, inner and outer sides, toes, and soles of the feet by using petrissage and effleurage massage techniques. Each massage application to these areas was repeated 3-5 times on average.

In the first stage, the researcher rubbed the soles of the participant’s feet before initiating the massage. In the second stage, the researcher made circular movements by applying pressure to the sole of the pregnant woman’s feet with her thumb and applied pressure to the foot with up and down movements by using the metacarpophalangeal joints with her hand fisted. In the third stage, the researcher squeezed the participant’s heel and ankle between her thumb and forefinger and then gave a massage to the areas by kneading. Then, the massage application was terminated by applying compressions and frictions to each knuckle of the fingers and points between the fingers and between the thumb and forefinger^29^. The researcher who performed the massage obeyed the COVID-19 pandemic measures of the hospital rules during the implementation of the intervention.

### Control group

No pharmacological or non-pharmacological method was applied to the participants in this group to reduce their insomnia and anxiety levels. They were treated within the routine hospital protocol.

The post-intervention scores of the experimental and control groups on the ISI and STAI were evaluated.

### Data analyses

Data were analyzed using the SPSS software package. Scale scores were found to have a normal distribution by using skewness and kurtosis values^30^. Categorical data were compared using the chi-square test. Data with normal distribution were evaluated with t-tests. Statistical significance was set at p<0.05.

## FINDINGS

A total of 71 preeclamptic pregnant women participated in the study, with an age of 34 years and 30.26 weeks gestation. The mean age of the women was 34.5 years in the control group and 34.02 years in the massage group (Table 1).

Table 2 presents the participants in the experimental and control groups on the baseline and post-intervention scores of both ISI and STAI. Participants in the massage group had significantly declined ISI and STAI total scores corrected for post-intervention (p<0.050, Table 2).

**Table 1.** Comparison of the sociodemographic characteristics of the women participating in the study.

	Experimental group (n=35)		Control group (n=36)		t*	p
Age, mean (SD), years	34.02±4.28		34.5±3.8		-0.491	0.625
					X^2^	p
Education level, n (%)						
Primary education	14	40	16	44.4	3.541	0.170
High school	21	60	17	47.3		
University	0		3	8.3		
Work status, n (%)						
Yes	4	11.8	1	2.8	2.029	0.170
No	31	88.6	35	97.2		
Spouse’s education level, n (%)						
Primary education	15	42.9	19	52.8		
High school	14	40.0	16	44.4	4.162	0.125
University	6	17.1	1	2.8		
Spouse’s work status, n (%)						
Yes	29	82.9	31	86.1		
No	6	17.1	5	13.9	0.144	0.479
Income status, n (%)						
Low	9	25.7	10	27.8		
Middle	26	74.3	23	63.9	3.223	0.200
High	0	0	3	8.3		
Family type, n (%)						
Nuclear	32	91.4	35	97.2	1.120	0.296
Extended	3	8.6	1	2.8		
The planned state of pregnancy, n (%)						
Yes	19	54.3	19	52.8	0.016	0.544
No	16	45.7	17	47.2		
Number of pregnancy, mean (SD)	2.11±0.96		2.25±1.07		0.006	0.580
Weeks of pregnancy, mean (SD), w	30.4±2.30		30.13±2.41		0.289	0.866

t*: independent-samples t-test. x^2^: chi-square test.

**Table 2.** Intra- and inter-group comparisons of the mean Insomnia Severity Index and state anxiety inventory scores obtained by the participants in the foot massage and control groups.

Variables	Classical Foot massage group (n=37) Mean±SD	Control group (n=37) Mean±SD	t*	p^2^
ISI-1	16.6±7.51	18.77±6.53	-1.301	0.198
ISI-2	12.45±5.74	18.8±6.44	-4.382	**0.000**
t**	10.201	-0.329		
p^1^	**0.000**	0.744		
STAI-1	37.97±5.39	37.08±6.42	0.630	0.531
STAI-2	33.4±6.41	39.19±8.31	-3.294	**0.002**
t**	2.047	-10.167		
p^1^	**0.000**	**0.000**		

t*: independent-samples t-test. t**: dependent-samples t-test. p_1_: comparison of intra-groups. p_2_: comparisons between groups. ISI-1: Insomnia Severity Index-Before massage; STAI-1: State Anxiety Inventory-Before massage; ISI-2: Insomnia Severity Index-After massage; STAI-2: State Anxiety Inventory-After massage. Bold indicates statistically significant p-value.

## DISCUSSION

The international and domestic literature was examined, and no studies on the reduction of insomnia and anxiety in preeclamptic pregnant women by using classical foot massage were found. In this study, the effect of classical foot massage applied to pregnant women diagnosed with PE and hospitalized due to insomnia and anxiety symptoms was investigated. Study results were discussed within the framework of the results of other studies on the effect of foot massage applied to groups of women with insomnia and anxiety. These findings are consistent with other studies in which insomnia and anxiety levels are reduced after classical foot massage intervention^19^ ^,^ ^20^ ^,^ ^21^ ^,^ ^31^ ^,^ ^32^. Classical foot massage was shown to be effective in decreasing insomnia and anxiety levels. In a randomized controlled trial by Eguchi et al., 52 participants were divided into two groups. In the study, participants in the experimental group were given a foot massage for a total of 12 times with 3 sessions in a week for 4 weeks. As a result of the research, it was determined that the anxiety levels of the foot massage group decreased statistically compared with the control group who did not receive the application^20^. Saatsaz et al., conducted a study with 156 primiparous pregnant women and evaluated post-cesarean section anxiety and pain. The anxiety levels of the groups were determined, and the mean STAI scores of the participants in the experimental groups were significantly lower than the scores of those in the control group^32^.

According to the results of a randomized controlled trial by Gökbulut et al., with 70 postmenopausal women, insomnia, anxiety, and fatigue symptoms of the participants who received foot massage every day for 1 week were reduced statistically significantly compared with the group that did not receive massage^21^. In an experimental study by Arslan et al., on the evaluation of the sleep quality of 90 female patients with hypertension, the participants were given foot and back massages 2 days in a week for 3 weeks. It was determined that the scores of women in both intervention groups were significantly lower than the scores of the participants in the control group^19^.

The study has several limitations. Regarding the first limitation, the sample size was small. The second limitation was that participants were informed that they could quit the research whenever they wanted as participation in the study was on a voluntary basis. This prolonged the data collection process and caused data loss. Also, participants were not informed about the use of classical foot massage in order not to create a placebo effect, which was the third limitation of the study.

## CONCLUSION

Various research results to date have supported the use of massage, which is among the traditional and alternative methods, in the treatment of diseases^13^ ^,^ ^17^ ^,^ ^18^ ^,^ ^19^ ^,^ ^21^. No complications affecting the efficiency and reliability of this method have been reported in the literature^13^ ^,^ ^17^ ^,^ ^18^ ^,^ ^19^ ^,^ ^21^. In this study, it was determined that the mean ISI and STAI-2 scores of preeclamptic pregnant women who received a classical foot massage decreased significantly during their hospital stay. Integrating classical foot massage into health approaches offered to preeclamptic pregnant women who stay in hospital can facilitate the use of classical foot massage by health professionals.
